# Psychodynamic Guided Self-Help for Adult Depression through the Internet: A Randomised Controlled Trial

**DOI:** 10.1371/journal.pone.0038021

**Published:** 2012-05-29

**Authors:** Robert Johansson, Sigrid Ekbladh, Amanda Hebert, Malin Lindström, Sara Möller, Eleanor Petitt, Stephanie Poysti, Mattias Holmqvist Larsson, Andréas Rousseau, Per Carlbring, Pim Cuijpers, Gerhard Andersson

**Affiliations:** 1 Department of Behavioural Sciences and Learning, Linköping University, Linköping, Sweden; 2 Psychiatric Clinic, University Hospital of Linköping, Linköping, Sweden; 3 Department of Psychology, Umeå University, Umeå, Sweden; 4 Department of Clinical Psychology and EMGO Institute, Vrije Universiteit, Amsterdam, The Netherlands; 5 Swedish Institute for Disability Research, Linköping University, Linköping, Sweden; 6 Department of Clinical Neuroscience, Psychiatry Section, Karolinska Institutet, Stockholm, Sweden; University of Missouri-Kansas City, United States of America

## Abstract

**Background and aims:**

Psychodynamic psychotherapy (PDT) is an effective treatment for major depressive disorder (MDD), but not all clients with MDD can receive psychotherapy. Using the Internet to provide psychodynamic treatments is one way of improving access to psychological treatments for MDD. The aim of this randomised controlled trial was to investigate the efficacy of an Internet-based psychodynamic guided self-help treatment for MDD.

**Methods:**

Ninety-two participants who were diagnosed with MDD according to the Mini-International Neuropsychiatric Interview were randomised to treatment or an active control. The treatment consisted of nine treatment modules based on psychodynamic principles with online therapist contact. The active control condition was a structured support intervention and contained psychoeducation and scheduled weekly contacts online. Both interventions lasted for 10 weeks. The primary outcome measure was the Beck Depression Inventory-II (BDI-II).

**Results:**

Mixed-effects model analyses of all randomised participants showed that participants receiving Internet-based PDT made large and superior improvements compared with the active control group on the BDI-II (between-group Cohen's *d* = 1.11). Treatment effects were maintained at a 10-month follow-up.

**Conclusions:**

Internet-based psychodynamic guided self-help is an efficacious treatment for MDD that has the potential to increase accessibility and availability of PDT for MDD.

**Trial Registration:**

Clinicaltrials.gov: NCT01324050

## Introduction

Major depressive disorder (MDD) is a major health problem, which lowers the quality of life for the individual and generates huge costs for society [1], [2]. Only about half of the 12-month cases in the USA were receiving treatment for MDD and only 18–25% were adequately treated [3]. Several forms of psychotherapy have been found to be effective in the treatment of MDD [4]. Among these, cognitive–behavioural therapy (CBT) has a strong empirical base [4].

Several studies have found that it is possible to deliver CBT as Internet-based guided self-help [5], and an increasing number of studies show that this treatment format can be as effective as face-to-face CBT for mild to moderate MDD and anxiety disorders [6]. Guided Internet treatments have provided a way to reach out to more patients in a manner that in most cases requires less therapist time [7].

Psychodynamic psychotherapy (PDT) is another psychological treatment that is effective for depression [8]. However, it is not known if it is possible to deliver PDT for MDD as guided self-help via the Internet. To our knowledge, no trial on Internet-delivered PDT has been published. It is important to examine if Internet-delivered PDT is effective, both from a theoretical and a practical point of view, since patients may prefer it above CBT. The aim of this study was to investigate the efficacy of a 10-week psychodynamic treatment for MDD, delivered in the form of guided self-help via the Internet. We compared the treatment to an active control condition that consisted of psychoeducation and scheduled support, also given for 10 weeks via the Internet. Significant within-group effects were expected for both conditions, but the effects for the treatment group were expected to be larger on measures of depression.

## Methods

### Ethics statement

The study was approved by the Regional Ethics Board of Linköping, Sweden. Signed informed consent was obtained from all participants via the online treatment platform.

### Participants and recruitment

The protocol for this trial and supporting CONSORT checklist are available as supporting information; see Checklist S1 and Protocol S1. Participants were recruited nationally through an advertisement in a major Swedish newspaper two weeks before the treatment began. Additional participants were recruited from a waiting list for another treatment trial for depression. The study was approved by the Regional Ethics Board of Linköping, Sweden. Written informed consent was obtained from all participants during the online screening. Inclusion criteria for the study were a) being at least 18 years old, b) having a total score in the range of 15 to 35 on the self-rated version of the Montgomery-Åsberg Depression Rating Scale (MADRS-S) [9], c) no assessed risk of suicidality (see below for details), d) if on medication, unchanged dosage of psychiatric medication during the three months preceding the screening, e) no concurrent psychological treatment, f) not having other primary disorders that needed different treatments or that could be affected negatively by the treatment, g) a diagnosis of MDD according to the DSM-IV, with current acute episode of depression or an episode in partial remission.

Applicants to the study were instructed to complete an online screening containing demographical questions and the outcome measures described below. A participant was contacted for a telephone-based diagnostic interview if he or she had completed the screening and met the initial inclusion criteria. In the telephone interview, diagnostic questions about depression and anxiety disorders were asked in addition to questions about use of medications and psychological treatments. Additionally, an assessment of suicidal ideation was conducted.

**Figure 1:**
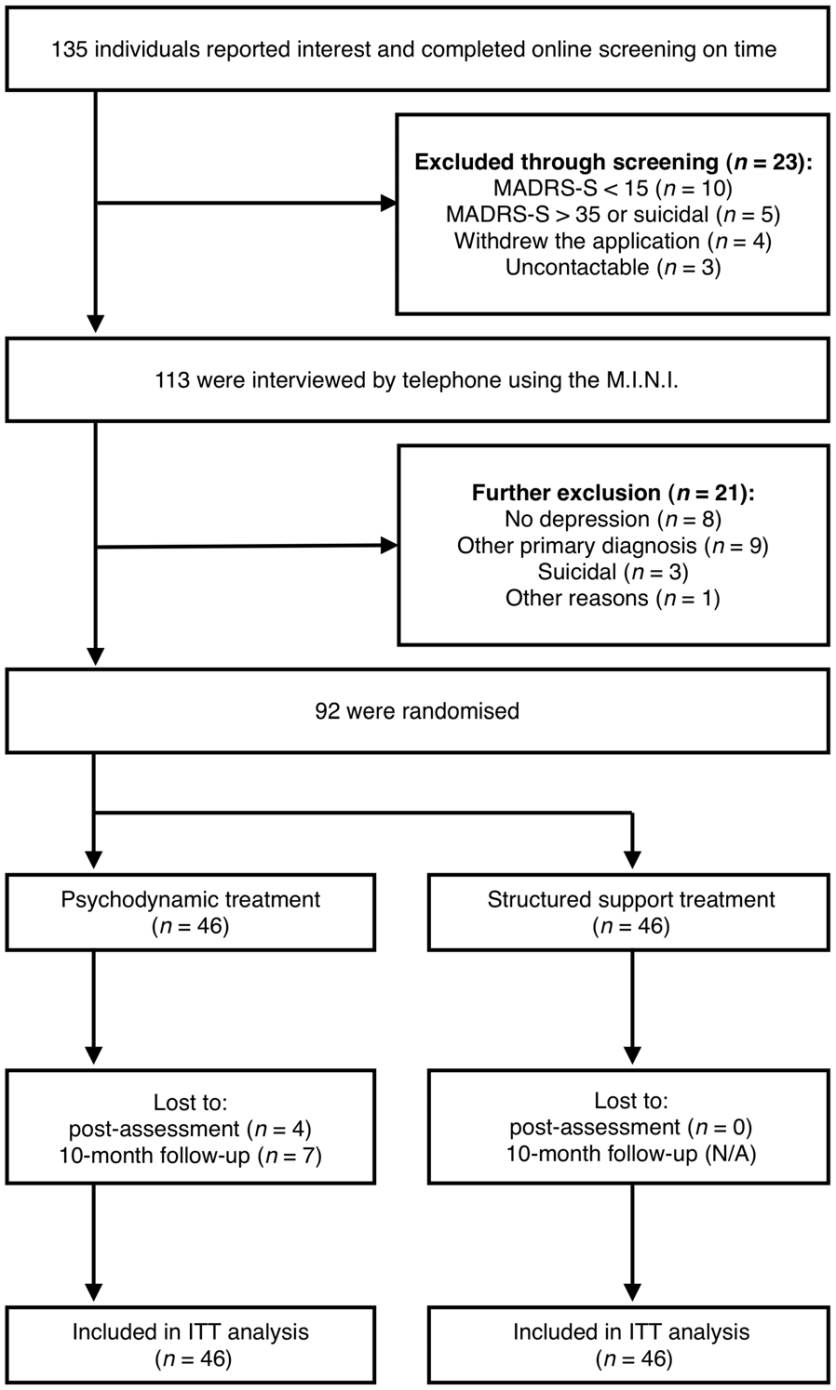
Participant flow and reasons for exclusion. Abbreviations: MADRS-S: Montgomery-Åsberg Depression Rating Scale-Self-rated version; M.I.N.I.: Mini-International Neuropsychiatric Interview; ITT: Intention-to-treat.

Six final semester-M.Sc. clinical psychology students who had been trained in the diagnostic procedures conducted the interviews. To ensure reliability and quality in the procedure, a psychiatrist was available for consultation during the entire assessment phase. Before a participant was included, the psychiatrist and the senior researcher reviewed the screening results and the interview protocol. Figure 1 shows the participant flow throughout the trial and reasons for exclusion. The demographic data are presented in Table 1.

**Table 1:** Demographic description of the participants at randomization.

		Psychodynamic treatment	Support treatment	Total
Gender	Female	37 (80.4%)	32 (69.6%)	69 (75.0%)
	Male	9 (19.6%)	14 (30.4%)	23 (25.0%)
Age	Mean (SD)	45.5 (15.2)	45.8 (12.8)	45.6 (14.0)
	Min-Max	22–73	21–72	21–73
Marital status	Married or co-habiting	31 (67.4%)	29 (63.0%)	60 (65.2%)
	Other	15 (32.6%)	17 (37.0%)	32 (34.8%)
Educational level	College or university, at least 3 years	29 (63.0%)	33 (71.7%)	62 (67.4%)
	College or university, shorter than 3 years	9 (19.6%)	6 (13.0%)	15 (16.3%)
	Other	6 (13.0%)	6 (13.0%)	12 (13.0%)
Employment status	Employed	32 (69.6%)	38 (82.6%)	70 (76.1%)
	Other	14 (30.4%)	8 (17.4%)	22 (23.9%)
Medication	Present	10 (21.7%)	13 (28.3%)	23 (25.0%)
	Prior experience	17 (37.0%)	9 (19.6%)	26 (28.3%)
	No experience	19 (41.3%)	24 (52.2%)	43 (46.7%)
Psychological treatment	Prior experience	29 (63.0%)	25 (54.3%)	54 (58.7%)
	No experience	17 (37.0%)	21 (45.7%)	38 (41.3%)
Depression	In acute episode	32 (69.6%)	28 (60.9%)	60 (65.2%)
	In partial remission	14 (30.4%)	18 (39.1%)	32 (34.8%)
Comorbidity	Social anxiety disorder	15 (32.6%)	14 (30.4%)	29 (31.5%)
	Generalized anxiety disorder	16 (34.8%)	11 (23.9%)	27 (29.3%)
	Panic disorder	5 (10.9%)	2 (4.3%)	7 (7.6%)
	Obsessive compulsive disorder	1 (2.2%)	1 (2.2%)	2 (2.2%)
	Post-traumatic stress disorder	3 (6.5%)	0 (0.0%)	3 (3.3%)
	Any anxiety disorder	26 (56.5%)	23 (50.0%)	49 (53.3%)

### Outcome measures

#### Primary outcome measure

The primary outcome measure was the Beck Depression Inventory-II (BDI-II) [10] that was administered pre-treatment, on a weekly basis during the entire treatment phase, at post-treatment and also 10 months after the treatment had ended.

#### Secondary outcome measures

Other outcome measures were collected at pre-treatment, post-treatment and at a 10-month follow-up. The results from the online screening were used as pre-treatment assessment. In addition to the BDI-II, measures of depression included the self-rated version of the Montgomery-Åsberg Depression Rating Scale (MADRS-S) [9] and the 9-item Patient Health Questionnaire Depression Scale (PHQ-9) [11]. Two measures of anxiety were used-the Beck Anxiety Inventory (BAI) [12] and the 7-item Patient Health Questionnaire Generalized Anxiety Disorder Scale (GAD-7) [13]. Finally, life quality was measured using the Quality of Life Inventory (QOLI) [14].

#### Clinician-administered measures

Psychiatric diagnoses (from the DSM-IV) were assessed using the Mini-International Neuropsychiatric Interview (M.I.N.I.) [15]. The M.I.N.I. is a diagnostic interview that, in contrast to several other diagnostic interviews, is completely structured, making it appropriate for other assessors than experienced psychiatrists [15]. At post-assessment, another structured telephone interview was conducted. The purpose of the interview was to give an estimation of global improvement, measured by the 7-point version of the Clinical Global Impression-Improvement (CGI-I) scale [16]. All interviews were conducted by the six psychology students described above, who at post-treatment were not blind to participant's condition. During the 10-month follow-up period, the participants were assessed with the CGI-I once again. All follow-up interviews were conducted by a final semester-M.Sc. clinical psychology student.

### Procedure

For those participants included in the study, the results from the online screening were used as pre-treatment assessment. The outcome measures that were collected pre-treatment were also collected at post-treatment and at follow-up. All measures used have been shown to have good psychometric properties, with internal consistencies of at least *α* = .79. Details of this can be found in the respective references of the outcome questionnaires. The measures were administered via the Internet, which has been shown to be a valid format for questionnaires regarding depression and anxiety [17], [18].

The participants were allocated to the psychodynamic treatment or to the active control condition in a 1∶1 ratio using block randomisation. An independent person, separate from the staff conducting the study, handled the randomisation using an online randomisation tool.

### Interventions

#### Psychodynamic treatment and therapists

The psychodynamic treatment was given as guided self-help, with minimal text-based guidance provided on a weekly basis [7]. In all, there were nine treatment modules, totalling 167 pages of text. Participants were given gradual access to the self-help modules and had continuous online support from a therapist using a secure online messaging system, similar to encrypted e-mail.

**Figure 2:**
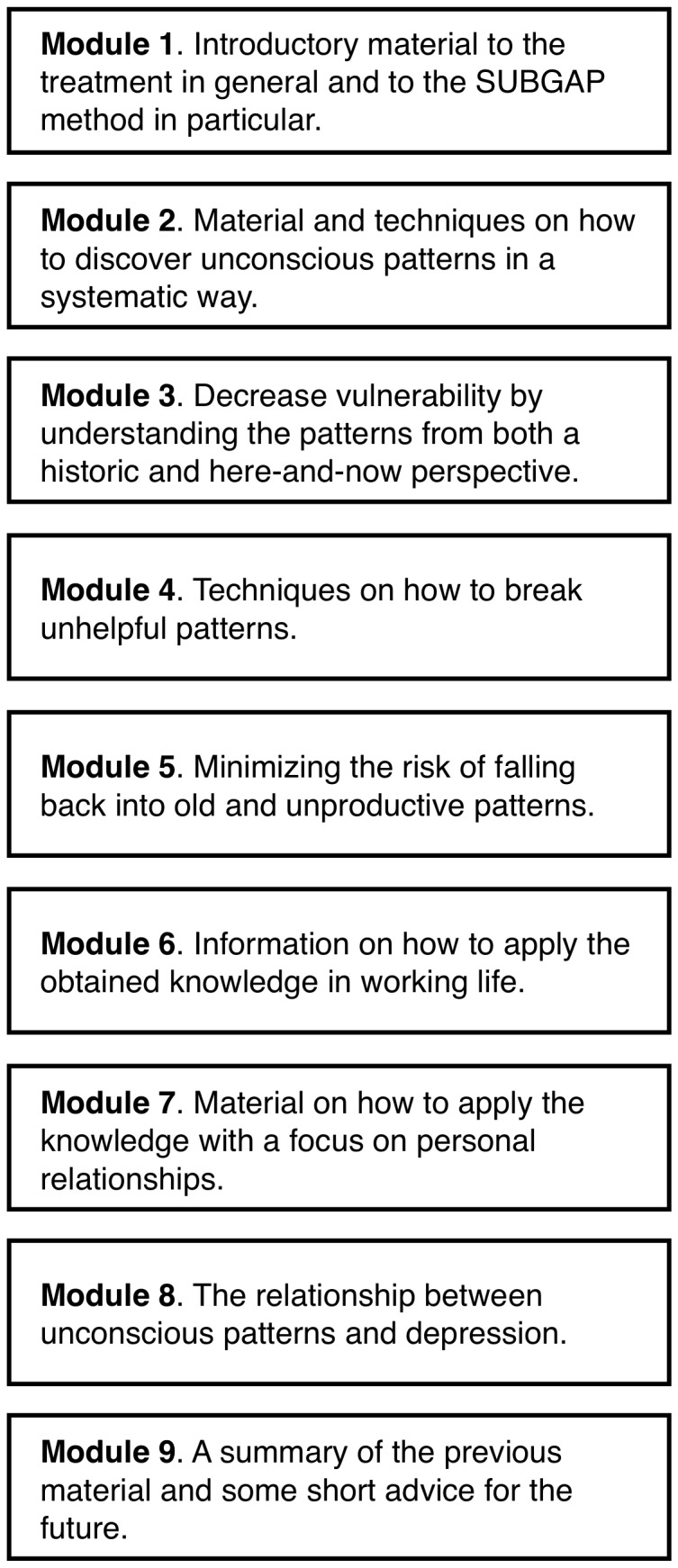
Description of the self-help modules in the psychodynamic treatment.

The treatment modules were largely derived from the self-help book Make the leap [19] that is based on psychodynamic principles. To make the material suitable for depression, the text was adapted and an extra chapter was written, which contained a psychodynamic understanding of how depression is developed and maintained [20]. The overall focus of the treatment was on teaching the client how to see and break unhelpful affective, cognitive and behavioural patterns. The treatment was called SUBGAP, which stands for (1) Seeing unconscious patterns that contribute to emotional difficulties, (2) Understanding these patterns, (3) Breaking such unhelpful patterns, and (4) Guarding Against Patterns and/or relapses [19]. A detailed description of the treatment is provided in Figure 2. All treatment modules ended with an encouragement for the participants to try out the SUBGAP strategies described in the particular module and write to the therapists about the experiences from this. The therapists gave feedback on the clients' experiences and administered the gradual access to the modules. In general, feedback was given on Mondays, but the therapists were available to answer additional questions within 24 hours.

#### Scheduled online supportive treatment

The group that served as the active control group received psychoeducation and scheduled online support delivered in the same online environment as the psychodynamic treatment. Similarly, this intervention lasted for 10 weeks. During the first week of support, all participants received text material on depression. The text was 15 pages long and contained general information about depression, including DSM-IV criteria, epidemiology and treatment alternatives. All participants were assigned to an individual therapist who provided the support. Every Monday the participants were contacted by the therapist and were asked questions about the previous week. The therapists were instructed to give support, but not to use any specific psychological techniques other than basic therapeutic skills such as empathic listening and asking further question to help the clients to express their experiences and emotions. In addition to the scheduled online support, the participants could send messages to the therapists at any time during the week and were then given response within 24 hours during weekdays. The intervention given to this group was similar to how non-directive supportive therapies have been described [4]. Non-directive supportive therapies have been shown to be effective for depression, but significantly less effective than other psychotherapies [4].

After the treatment period had ended, the participants in the support group were crossed over to treatment. They could then choose between the psychodynamic treatment and a previously developed CBT treatment [21]. The results from this treatment period are, however, outside the scope of this study.

#### Therapists

The therapists were six final-semester students from a five-year M.Sc. clinical psychologist programme. All therapists had completed their clinical training as well as 16 weeks of internship. Each therapist was responsible for 7 to 8 treatment participants from the treatment group and an equal number of participants from the control group. Therapists were randomly allocated to participants, with the restriction of not having more than 8 participants from each group. For the entire duration of the study the therapists received continuous supervision from an experienced psychotherapist with psychodynamic orientation, who had previous experience of the psychodynamic treatment manual. Typically, supervision consisted of examination of specific online interactions as well as more general therapeutic issues. Clients from both groups were discussed during supervision. During treatment, the therapists also had the possibility to consult the psychiatrist, e.g. on medication issues or if a participant expressed suicidal ideation.

### Data analyses

All analyses were performed using SPSS 19 (SPSS, Inc., Chicago, IL). Independent *t*-tests and *χ^2^*-tests were used to test for group differences in demographics, pre-treatment data and in clinical significant improvement. Differences between the psychodynamic treatment and the structured support were primarily investigated by modelling interaction effects of group and time. In order to adhere to the intention-to-treat principle, the continuous outcome variables were analysed using mixed effects models, given their ability to handle missing data [22]. All analyses used Maximum Likelihood estimation. Random intercept models were selected for all measures except for the BDI-II. Group, time and their interaction were included as predictors in these models. For the BDI-II, where weekly measures were available, several models were compared using available information criteria, and the model with best fit was chosen. This model included a fixed linear effect of time with a random intercept and slope. The covariance between the random intercept and slope was not significant, so it was not included in the model. Error terms across time were modelled with a first-order autoregressive covariance structure with heterogeneous variances. Differences in average rates of growth between the two groups were examined by a fixed effects interaction between group and time. Between-group differences at post-treatment were analysed using independent *t*-tests. Power analysis indicated an 89% chance of detecting a between-group effect size of *d* = 0.60 (*α* level = 0.05).

To investigate recovery after treatment and at follow-up, the BDI-II was used. Recovery was defined as a post-treatment BDI-II score ≤10. This definition is in line with previous clinical trials on depression (e.g. [23], [24]). Participants who did not provide post-treatment data or follow-up data were classified as non-recoverers.

Within- and between-group effect sizes (Cohen's *d*) were calculated by dividing the differences in means by the pooled standard deviations [25]. The between-group effect sizes can be interpreted as follows: an effect size in the range of 0.20–0.49 is small, while 0.50–0.79 is moderate, and an effect size over 0.80 is large [26].

## Results

The treatment group and the support group did not differ significantly on any of the pre-treatment measures (all *t*'s<1.47, all *p*'s>.14). Additionally, there were no significant differences between the groups on any demographic data or current/past treatment with medication and/or psychological treatment. Results from the mixed-effects model analyses are presented below. Means, standard deviations and effect sizes within and between groups for all self-report measures are presented in Table 2.

**Table 2:** Means, SDs and effect sizes (Cohen's d) for measures of depression, anxiety and quality of life.

	Mean (*SD*)			Effect size. *d* (95% CI)		
Outcome measure	Pre- treatment	Post-treatment	10-month follow-up	Between-group, post- treatment	Within-group, pre-post- treatment	Within-group, pre - 10-month follow-up
BDI-II						
Psychodynamic treatment	26.54 (5.8)	11.48 (7.8)	10.38 (9.6)	1.11 (0.67–1.56)	2.18 (1.49–2.86)	1.94 (1.41–2.47)
Structured support treatment	26.33 (6.7)	20.22 (7.8)			0.84 (0.46–1.21)	
MADRS-S						
Psychodynamic treatment	23.07 (4.6)	12.50 (7.8)	11.23 (9.1)	0.86 (0.43–1.30)	1.56 (1.09–2.04)	1.52 (1.05–1.99)
Structured support treatment	23.48 (5.1)	18.61 (6.4)			0.84 (0.44–1.25)	
PHQ-9						
Psychodynamic treatment	12.61 (4.1)	6.24 (5.0)	5.00 (5.4)	0.95 (0.51–1.39)	1.46 (0.90–2.02)	1.64 (1.05–2.23)
Structured support treatment	13.30 (4.3)	10.87 (4.8)			0.54 (0.21–0.86)	
GAD-7						
Psychodynamic treatment	8.65 (3.4)	5.29 (4.0)	4.10 (4.1)	0.52 (0.09–0.94)	0.97 (0.58–1.36)	1.20 (0.71–1.69)
Structured support treatment	8.50 (4.2)	7.61 (4.9)			0.2 (−0.14–0.53)	
BAI						
Psychodynamic treatment	20.96 (9.5)	12.00 (8.9)	9.54 (10.0)	0.15 (−0.27–0.57)	0.97 (0.66–1.29)	1.15 (0.74–1.55)
Structured support treatment	19.39 (10.2)	13.35 (8.8)			0.63 (0.37–0.88)	
QOLI						
Psychodynamic treatment	0.35 (1.5)	1.18 (1.7)	1.65 (2.0)	0.59 (0.16–1.02)	0.48 (0.22–0.75)	0.69 (0.35–1.03)
Structured support treatment	−0.08 (1.3)	0.23 (1.5)			0.22 (−0.04–0.48)	

Abbreviations: BDI-II: Beck Depression Inventory-II; MADRS-S: Montgomery-Åsberg Depression Rating Scale-Self-rated version; PHQ-9: 9-item Patient Health Questionnaire Depression Scale; GAD-7: Patient Health Questionnaire Generalized Anxiety Disorder Scale; BAI: Beck Anxiety Inventory; QOLI: Quality of Life Inventory.

### Attrition and adherence

Four out of 92 participants (4.3%) did not provide post-treatment data. Three out of these and two additional participants (totalling 5.4%) were unreachable for the telephone interview and were classified as unimproved according to the CGI-I. In the 10-month follow-up, 39 participants from the treatment group (84.8%) provided data on the self-report measures and 38 (82.6%) were reached for the telephone interview. Once again, those unreachable were classified as unimproved on the CGI-I. Follow-up data from the control group was also collected, but is reported elsewhere.

The number of completed treatment modules was used as a measure of adherence in the treatment group. A module was considered to be finished only if the weekly discussion of the module was sent to the therapist. Two participants (4.3%) did not start the treatment at all. One (2.1%) stopped after the first module, one after the third, three (6.5%) after the fourth, two after the fifth and one after the sixth. In total, 36 out of 46 participants (78.3%) in the treatment group finished all modules.

### Primary outcome measure

**Figure 3:**
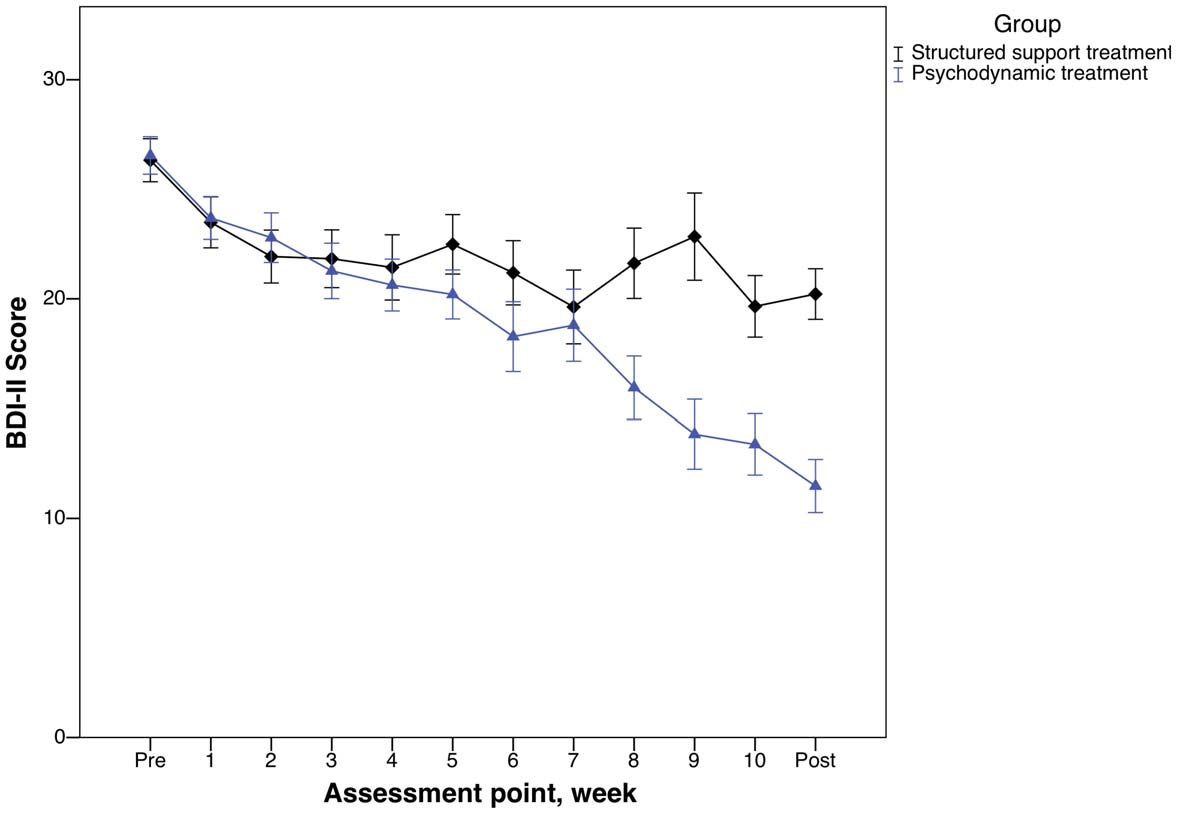
Weekly change on the Beck Depression Inventory-II during treatment and Beck Depression Inventory-II scores at each assessment point.

As illustrated in Figure 3, the psychodynamic treatment group displayed continuous within-group improvements throughout the trial on the BDI-II. As seen in Table 2, the effect size between the groups at post-treatment was large (Cohen's *d* = 1.11). There was a substantial within-group effect size in the structured support group as well, indicating an effect in both groups. Mixed-effect model analyses showed a significant interaction effect of group and time on the BDI-II (*F*(1, 109.8) = 37.2, *p*<.001). The post hoc *t*-test was significant (*t*(86) = 5.23, *p*<.001).

### Secondary outcome measures

#### Measures of depression

As seen in Table 2, there were large between-group effect sizes on the MADRS-S and the PHQ-9 as well. Mixed models analyses showed significant interaction effects of group and time (*F*(1, 92.1) = 15.2, *p*<.001 and *F*(1, 90.7) = 13.1, *p*<.001, for the MADRS-S and the PHQ-9 respectively). Post-hoc *t*-tests were all significant (all *t*'s>4.05, all *p*'s<.001).

#### Measures of anxiety and quality of life

On the GAD-7, the mixed models analysis showed a significant interaction effect of group and time (*F*(1, 90.8) = 6.92, *p*<.05). The post-hoc *t*-test was significant (*t*(86) = 2.42, *p*<.05). The mixed models analyses conducted on the BAI and the QOLI did not reveal any significant interaction effects, although the *p*-values were close to significant (*F*(1, 90.0) = 3.28, *p* = .074 and *F*(1, 88.1) = 3.07, *p* = .083, for the BAI and the QOLI respectively. As seen in Table 2, both groups had substantial within-group effects on the BAI.

### Recovery after treatment

There were differences in recovery rates (post-treatment BDI-II score of ≤10) between the groups at post-treatment. The intervention group had a significantly larger proportion of participants who recovered after treatment (*n* = 16; 34.8%) than the control group (*n* = 4; 8.7%), *χ^2^*(N = 92, df = 1) = 9.2, *p*<.01. At the 10 month follow-up, 25 out of 46 participants (54.3%) from the treatment group had recovered.

### Clinical global improvement and adverse events

Of the 92 participants randomised, 42 from the treatment group and 45 from the control group were reached for a post-treatment telephone interview that gave an estimate of the clinical global improvement on the CGI-I [16]. Unreachable participants were classified as unimproved. In the treatment group, 24 participants (52.2%) were much or very much improved while this was only true for 13 (28.3%) in the support group. The difference was significant, *χ^2^*(N = 92, df = 1) = 5.47, *p*<.05. Two participants from each group were minimally worse and one from the control group was much worse and therefore classified as adverse events. Of the two treatment group participants, one did not begin treatment, while the other reported feeling worse since she did not understand how to make use of the material. In the 10-month follow-up interview, 27 participants (58.7%) were classified as much or very much improved.

### Therapist time

As expected, the average therapist time per participant and week was larger in the treatment group compared to the support group (13.2 minutes compared to 4.5 minutes, *t*(90) = 8.57, *p*<.001). Importantly, this was the time that the therapists were logged on to the online treatment platform, i.e., not including time for between-session reflection.

## Discussion

This trial tested the efficacy of a 10-week psychodynamic treatment for depression, given as guided self-help over the Internet. The treatment was compared to an active control in the form of a supportive treatment that contained psychoeducation and scheduled online contacts. The main finding was that participants who received psychodynamic treatment improved more than those who received the scheduled support. Between-group effect sizes on measures of depression were large and the treatment effects were maintained at the 10-month follow-up. The results indicate that psychodynamic guided self-help is effective in the treatment of depression and that it is possible to deliver psychodynamic therapy via the Internet.

When developing the psychodynamic guided self-help treatment, we made the assumption that the core ingredients of psychodynamic theory could be retained. Internet-based treatments still involves therapist contact, albeit different from face-to-face treatment. The treatment manual aimed to preserve the psychodynamic principles and was derived from a book by an experienced psychoanalyst [19].

One fundamental aspect of psychodynamic psychotherapy is the therapeutic relationship [27]. A therapist contact of 10 to 15 minutes per week and client may seem insufficient to establish a strong relationship. However, the therapists acted in a personal manner and aimed to build a strong therapeutic alliance, e.g. by using supportive techniques and by answering any messages within 24 hours. Although alliance was not directly measured in this study, it is known that a strong therapist-client alliance can be established in Internet-based treatments [28], even when the therapist contact is brief [29]. Consequently, this treatment seems to be more supportive than expressive, using the supportive-expressive distinction established by Luborsky [30]. Since all communication in the guided self-help treatment was in a medium similar to e-mail, it was neither possible for the therapists to do any in-session exploration of the clients' affective experience nor any interpretation of transference processes, which are interventions which distinguishes psychodynamic face-to-face treatments from e.g. cognitive-behavioural therapy [27].

The psychodynamic guided self-help treatment had a continuous focus on identifying affective, cognitive and interpersonal patterns that had led to problematic behaviour for the client. Furthermore, several treatment modules had an emphasis on past experiences as well as on interpersonal experiences. These are also typical elements of psychodynamic therapy [27]. While not including homework in the classical sense, the treatment modules contained descriptions of techniques that the participants were encouraged to apply in their own lives. This focus and encouragement of activities outside-of-session is traditionally associated with cognitive-behavioural treatments [31], but in actual practice many psychodynamic practitioners tend to use homework [32].

Ten weeks are indeed an unusually short duration for a psychodynamic treatment, making it reasonable to question if such a treatment is possible to conduct in such a short time. It is possible that the decrease in symptoms may have continued for the treatment group if the treatment had been longer than 10 weeks. However, results from the Sheffield studies have shown comparable effects between 8- and 16-week psychodynamic therapy, indicating that psychodynamic treatment indeed can be as short as 10 weeks and that longer therapy may not necessarily result in larger effects [33].

There are limitations that must be addressed. First, the participants were recruited from the community and we cannot be sure that this treatment would work in a clinical setting, e.g. an outpatient psychiatric facility. However, mean depression severity as measured by the BDI-II at intake (*M* = 26.51) is close to the limit of 29 that Beck proposes for severe depression [10], and the fact that a majority of the participants had a comorbid anxiety disorder seems to parallel clinical reality [34]. A second related concern was the large number of participants who had college- or university level education. This might bias generalizability of the results, since it is possible that guided self-help is especially well suited for educated clients. However, there are data indicating that 50% of patients seeking psychotherapy have some college education [35] and that educated patients may be more inclined to seek help for mental health problems [36].

As a third limitation, we allowed the participants to have a parallel treatment of medication if stable for three months, which was true for 25% of the participants. This is common procedure in previous trials from our group (e.g., [21]). Medication status was unrelated to change on the BDI-II in this trial, but we cannot rule out that there was a small additive effect of medication in the sample.

A fourth limitation that needs to be addressed concerns the therapists in the study who all were psychologists in training, albeit during the last semester of training in a five year program and under regular supervision. There are some indications in the literature that students may be less effective as therapists when conducting face-to-face therapy [37]. Therefore it is not possible to rule out that experienced psychotherapists would have performed even better. However, this can be contrasted with recent indications that a computer technician can conduct Internet-based CBT as good as a clinician [38]. These recent results call for further research on who can conduct guided self-help treatments.

A related concern is that psychologists in training conducted all diagnostic interviews. Although the M.I.N.I. was designed to be administered by non-experts [15], there is a possibility that the study sample could have been more adequately defined. The interviewers were all trained in the procedure, but there were no procedures to ensure inter-rater reliability, which is a further limitation. The lack of blinding is also an important limitation, as it may have biased the results on the clinical interviews. Future studies should make use of more formal training in diagnostic procedures and blinding of assessors.

There are some implications of this study in addition to the aforementioned. While a significant number of randomised controlled trials have been conducted investigating the effects of psychodynamic psychotherapy, there has been a recent call for high quality, adequately powered trials targeting specific disorders [39]. As pointed out by Connolly Gibbons, Crits-Christoph and Hearon [40], there is no trial on depression that demonstrates the superiority of a manualised psychodynamic monotherapy over a control condition or another treatment. In that respect, one implication of this study is that it adds to the empirical base of psychodynamic treatment of depression. This study indicates that psychodynamic treatment might be on par with other treatments for depression. However, before any further conclusions are drawn, the results need to be replicated in other sites. In addition, direct comparisons to other established bona fide treatments (e.g., cognitive–behavioural therapy) need to be conducted.

This study also shows that psychodynamic psychotherapy is possible to deliver as guided self-help, which naturally has implications for dissemination of psychotherapy in general. There is a lack of psychodynamic therapy in other treatment formats than individual and group. Using the Internet format of delivery makes it possible to reach individuals who lack access to therapists nearby, or who simply do not want to meet face-to-face.

The availability of psychodynamic treatments that are possible to deliver via the Internet may also influence therapists' attitudes towards Internet treatments. There are some indications that CBT therapists may have a more positive attitude towards Internet-based treatments than therapists with psychoanalytic orientation [41], [42]. Thus, one implication of this study is that psychodynamic/psychoanalytic therapists in general may become more positive towards this treatment modality, which in the long run could mean that more patients get access to evidence-based treatments.

In summary, this trial demonstrates the efficacy of a treatment that presents psychodynamic principles in self-help text, accompanied by encrypted e-mail contact with a therapist working in a supportive fashion and aiming to build a strong alliance. The findings from this study add to the empirical base of psychodynamic treatments for depression and indicate that it is indeed possible to conduct psychodynamic treatment as guided self-help via the Internet.

## Supporting Information

Checklist S1 **CONSORT checklist.**
(DOC)

Protocol S1 **Original study protocol, translated to English.**
(DOC)
